# Bis(2-amino-4-methyl­pyrimidin-3-ium) *trans*-diaqua­bis­(pyrazine-2,3-di­car­boxylato)cobaltate(II) hexa­hydrate

**DOI:** 10.1107/S1600536810037736

**Published:** 2010-09-30

**Authors:** Hossein Eshtiagh-Hosseini, Marek Necas, Nafiseh Alfi, Masoud Mirzaei

**Affiliations:** a Department of Chemistry, School of Sciences, Ferdowsi University of Mashhad, Mashhad 917791436, Iran; b Department of Chemistry, Faculty of Science, Masaryk University, Kamenice 5, Brno, 625 00, Czech Republic

## Abstract

In the crystal structure of the mononuclear title compound, (C_5_H_8_N_3_)_2_[Co(C_6_H_2_N_2_O_4_)_2_(H_2_O)_2_]·6H_2_O or (ampymH)_2_[Co(pyzdc)_2_(H_2_O)_2_]·6H_2_O (ampym = 2-amino-4-methyl pyrimidine, pyzdcH_2_ = pyrazine-2,3-dicarb­oxy­lic acid), the Co^II^ ion is hexa­coordinated by two (pyzdc)^2−^ groups in the equatorial plane and two water mol­ecules in axial positions, giving an N_2_CoO_4_ bound set. The (pyzdc)^2−^ anion acts as a bidentate ligand through one carboxyl­ate group O atom and pyrazine ring N atom. There are diverse N—H⋯ O and O—H⋯O and O—H⋯N hydrogen-bonding inter­actions, which lead to the formation of a three-dimensional supra­molecular architecture. Off-set or slipped π–π stacking inter­actions are also observed between adjacent pyrimidine rings with face-to-face distances of 3.6337 (9) Å.

## Related literature

For the pyzdcH_2_ ligand, see: Aghabozorg *et al.* (2008 ▶). For the crystal structure of pyrazine-2,3-dicarb­oxy­lic acid (pyzdcH_2_), see: Takusagawa & Shimada (1973 ▶). For complexes of pyzdcH_2_ with zinc and manganese, see: Eshtiagh-Hosseini *et al.* (2010*a* ▶,*b* ▶,*c* ▶,*d* ▶,*e* ▶). The six uncoordinated water mol­ecules increase the number of hydrogen bonds and lead to the formation of (H_2_O)_n_ clusters throughout the crystal, see: Aghabozorg *et al.* (2010 ▶).
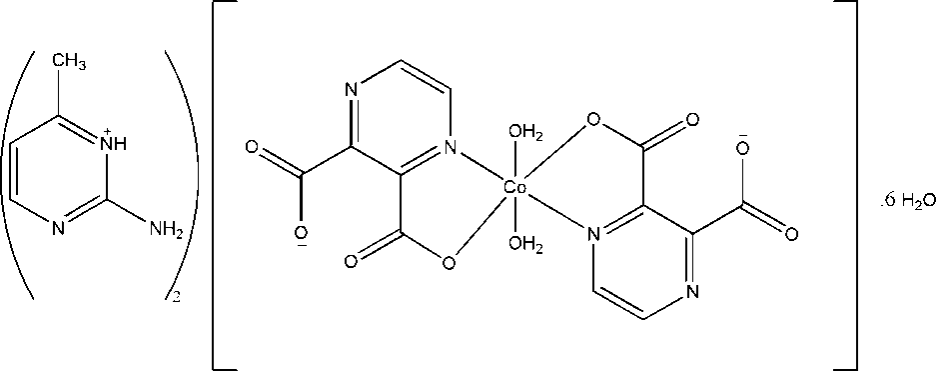


## Experimental

### 

#### Crystal data

(C_5_H_8_N_3_)_2_[Co(C_6_H_2_N_2_O_4_)_2_(H_2_O)_2_]·6H_2_O
*M* *_r_* = 755.54
Triclinic, 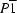

*a* = 6.5880 (4) Å
*b* = 8.0591 (5) Å
*c* = 15.0285 (8) Å
α = 98.085 (5)°
β = 96.940 (4)°
γ = 91.261 (5)°
*V* = 783.58 (8) Å^3^
*Z* = 1
Mo *K*α radiation
μ = 0.64 mm^−1^
*T* = 120 K
0.40 × 0.40 × 0.20 mm

#### Data collection

Oxford Diffraction Xcalibur diffractometer with Sapphire2 detector
Absorption correction: multi-scan (*CrysAlis RED*; Oxford Diffraction, 2009 ▶) *T*_min_ = 0.903, *T*_max_ = 1.000
5733 measured reflections
2758 independent reflections
2428 reflections with *I* > 2σ(*I*)
*R*_int_ = 0.011

#### Refinement

*R*[*F* ^2^ > 2σ(*F* ^2^)] = 0.025
*wR*(*F* ^2^) = 0.064
*S* = 1.04
2758 reflections
256 parameters
H atoms treated by a mixture of independent and constrained refinement
Δρ_max_ = 0.30 e Å^−3^
Δρ_min_ = −0.40 e Å^−3^

### 

Data collection: *CrysAlis CCD* (Oxford Diffraction, 2009 ▶); cell refinement: *CrysAlis RED* (Oxford Diffraction, 2009 ▶); data reduction: *CrysAlis RED*; program(s) used to solve structure: *SHELXS97* (Sheldrick, 2008 ▶); program(s) used to refine structure: *SHELXL97* (Sheldrick, 2008 ▶); molecular graphics: *DIAMOND* (Crystal Impact, 2009 ▶); software used to prepare material for publication: *publCIF* (Westrip, 2010 ▶).

## Supplementary Material

Crystal structure: contains datablocks I, New_Global_Publ_Block. DOI: 10.1107/S1600536810037736/vm2043sup1.cif

Structure factors: contains datablocks I. DOI: 10.1107/S1600536810037736/vm2043Isup2.hkl

Additional supplementary materials:  crystallographic information; 3D view; checkCIF report

## Figures and Tables

**Table 1:** Hydrogen-bond geometry (Å, °)

*D*—H⋯*A*	*D*—H	H⋯*A*	*D*⋯*A*	*D*—H⋯*A*
N3—H3*A*⋯O3^i^	0.88	1.77	2.6487 (17)	172
N5—H5*C*⋯O21^ii^	0.88	1.96	2.8350 (18)	172
N5—H5*D*⋯O4^i^	0.88	1.96	2.8310 (18)	171
O21—H21*B*⋯O4^iii^	0.81 (2)	1.97 (3)	2.7777 (18)	175 (2)
O21—H21*A*⋯O1	0.85 (3)	1.86 (3)	2.7093 (18)	177 (2)
O5—H5*B*⋯O22^iv^	0.77 (3)	2.02 (3)	2.784 (2)	172 (2)
O5—H5*A*⋯O23^v^	0.83 (3)	1.88 (3)	2.706 (2)	173 (3)
O23—H23*B*⋯O3^vi^	0.78 (3)	2.06 (3)	2.8302 (19)	172 (3)
O22—H22*B*⋯O21	0.83 (3)	1.97 (3)	2.794 (2)	177 (3)
O23—H23*A*⋯O22	0.85 (3)	2.13 (3)	2.959 (2)	165 (2)
O22—H22*A*⋯N1^vii^	0.79 (3)	2.59 (3)	3.152 (2)	130 (2)
O22—H22*A*⋯N4^vi^	0.79 (3)	2.68 (3)	3.242 (2)	130 (2)

Symmetry codes: (i) 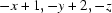; (ii) 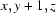; (iii) 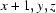; (iv) 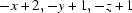; (v) 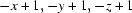; (vi) 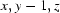; (vii) 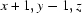.

**Fig. 1.**
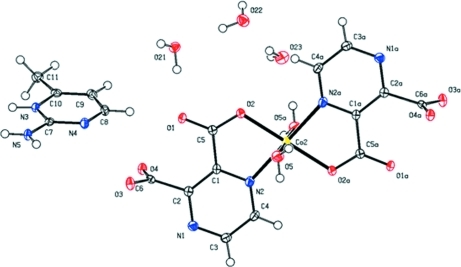
Molecular structure of (ampymH)2[Co(pyzdc)2(H2O)2].6H2O compound. Ellipsoids are drawn at the 50% probability level.

**Fig. 2.**
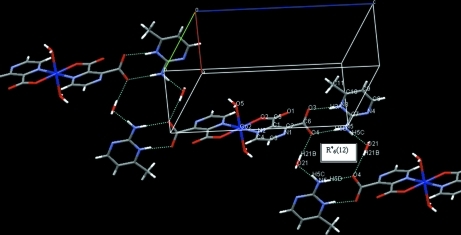
Schematic representation of the present R22(8) and R46(12) graph-sets in the crystalline network.

**Fig. 3.**
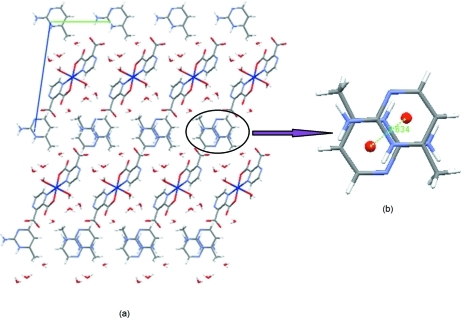
(a) Packing diagram along the a-axis. (b) The off-set or slipped π -π stacking interactions between aromatic rings of two (ampymH)+ fragments.

## supplementary crystallographic information

### Comment

Many organic aromatic ligands and metal ions may aggregate into supramolecular networks using coordination and hydrogen bonds and π –π stacking interactions. Metal pyrazine-(di)carboxylates may possess versatile structural motifs, which finally aggregate to various supramolecular architectures with interesting properties. For the first time, Takusagawa & Shimada (1973) determined the structure of pyzdcH_2_. Until now, several proton transfer compounds with pyzdcH_2_ have been synthesized by our research group such as, (ampyH)_2_[*M*(pyzdc)_2_(H_2_O)_2_].6H_2_O (*M* = Co (1), Cu, Zn and ampy = 2-amino-4-methyl pyridine) (Eshtiagh-Hosseini *et al.*, 2010*b*,*c**d*). Continuing our previous work on the syntheses of coordination compounds *via* proton transfer mechanism, we planned the reaction between pyzdcH_2_, ampym and cobalt^II^ chloride in order to provide a new coordination compound containing a proton transfer ligand. The title compound, (ampymH)_2_[Co(pyzdcH)_2_(H_2_O)_2_].6H_2_O, is analogous to previously synthesized compound 1 in which ampy was replaced by ampym (Fig. 1). The equatorial plane is occupied by two (pyzdc)^2- ^ligands coordinating through the pyrazine nitrogen and one oxygen of the deprotonated carboxylate groups. The two coordinated water molecules occupy the axial plane. This compound consists of an anionic moiety, *trans*-[Co(pyzdc)_2_(H_2_O)_2_]^2-^ complex, counter-ions, (ampymH)^+^, and six uncoordinated water molecules. The Co—O and Co—N bond distances related to the (pyzdc)^2- ^ligand are 2.0445 (12) Å, and 2.1086 (14) Å, respectively. The intermolecular forces between the anionic, cationic parts, and uncoordinated water molecules consist of hydrogen bonding interactions. The hydrogen bond interactions cause further stabilization for crystalline network using two types of graph-sets namely *R*^2^_2_(8) and *R*^4^_6_(12) (Fig. 2). Indeed, the arrangement of anionic parts in the network resulted in the creation of anionic holes for entering cationic parts; this arrangement results in off-set or slipped π -π stacking interactions with the distance of 3.6337 (9) Å between the centroids of the rings (Fig. 3). Moreover, six uncoordinated water molecules increase the number of hydrogen bonds in the crystalline network and lead to the formation of (H_2_O)_n_ clusters throughout the crystalline network (Aghabozorg *et al.* 2010).

### Experimental

A solution of pyzdcH_2_ (0.60 mmol, 0.10 g), ampym (1.2 mmol, 0.13 g), and CoCl_2_.6H_2_O (0.02 mmol, 0.05 g) at 333 K lead to formation of (ampymH)_2_[Co(pyzdc)_2_(H_2_O)_2_].6H_2_O orange block crystals after slow evaporation of solvent at room temperature.

### Refinement

Carbon and nitrogen bound hydrogen atoms were positioned geometrically and refined as riding using standard *SHELXTL* constraints, with their *U*_iso_ set to either 1.2*U*_eq_ or 1.5*U*_eq_ (methyl) of their parent atoms. The C-H distances were set to 0.95 and 0.98 \%A for aromatic and methyl groups, respectively, the N-H distances used were 0.88 \%A. Oxygen bound hydrogen atoms were located in a difference Fourier map and refined isotropically.

### Crystal data

**Table:**

(C_5_H_8_N_3_)_2_[Co(C_6_H_2_N_2_O_4_)_2_(H_2_O)_2_]·6H_2_O	*Z* = 1
*M**_r_* = 755.54	*F*(000) = 393
Triclinic, *P*1	*D*_x_ = 1.601 Mg m^−^^3^
Hall symbol: -P 1	Mo *K*α radiation, λ = 0.71073 Å
*a* = 6.5880 (4) Å	Cell parameters from 4417 reflections
*b* = 8.0591 (5) Å	θ = 3.1–27.6°
*c* = 15.0285 (8) Å	µ = 0.64 mm^−^^1^
α = 98.085 (5)°	*T* = 120 K
β = 96.940 (4)°	Plate, pale orange
γ = 91.261 (5)°	0.40 × 0.40 × 0.20 mm
*V* = 783.58 (8) Å^3^	

### Data collection

**Table:**

Oxford Diffraction Xcalibur diffractometer with Sapphire2 (large Be window) detector	2758 independent reflections
Radiation source: Enhance (Mo) X-ray Source	2428 reflections with *I* > 2σ(*I*)
graphite	*R*_int_ = 0.011
Detector resolution: 8.4353 pixels mm^-1^	θ_max_ = 25.0°, θ_min_ = 3.1°
ω scan	*h* = −7→7
Absorption correction: multi-scan (*CrysAlis RED*; Oxford Diffraction, 2009)	*k* = −9→9
*T*_min_ = 0.903, *T*_max_ = 1.000	*l* = −17→17
5733 measured reflections	

### Refinement

**Table:**

Refinement on *F*^2^	Primary atom site location: structure-invariant direct methods
Least-squares matrix: full	Secondary atom site location: difference Fourier map
*R*[*F*^2^ > 2σ(*F*^2^)] = 0.025	Hydrogen site location: inferred from neighbouring sites
*wR*(*F*^2^) = 0.064	H atoms treated by a mixture of independent and constrained refinement
*S* = 1.04	*w* = 1/[σ^2^(*F*_o_^2^) + (0.0279*P*)^2^ + 0.458*P*] where *P* = (*F*_o_^2^ + 2*F*_c_^2^)/3
2758 reflections	(Δ/σ)_max_ < 0.001
256 parameters	Δρ_max_ = 0.30 e Å^−^^3^
0 restraints	Δρ_min_ = −0.40 e Å^−^^3^

### Special details

**Table:**

Geometry. All e.s.d.'s (except the e.s.d. in the dihedral angle between two l.s. planes) are estimated using the full covariance matrix. The cell e.s.d.'s are taken into account individually in the estimation of e.s.d.'s in distances, angles and torsion angles; correlations between e.s.d.'s in cell parameters are only used when they are defined by crystal symmetry. An approximate (isotropic) treatment of cell e.s.d.'s is used for estimating e.s.d.'s involving l.s. planes.
Refinement. Refinement of *F*^2^ against ALL reflections. The weighted *R*-factor *wR* and goodness of fit *S* are based on *F*^2^, conventional *R*-factors *R* are based on *F*, with *F* set to zero for negative *F*^2^. The threshold expression of *F*^2^ > σ(*F*^2^) is used only for calculating *R*-factors(gt) *etc*. and is not relevant to the choice of reflections for refinement. *R*-factors based on *F*^2^ are statistically about twice as large as those based on *F*, and *R*- factors based on ALL data will be even larger.

### Fractional atomic coordinates and isotropic or equivalent isotropic displacement parameters (Å^2^)

**Table:**

	*x*	*y*	*z*	*U*_iso_*/*U*_eq_	
Co2	0.5000	0.5000	0.5000	0.01426 (11)	
O1	0.58615 (17)	0.62856 (14)	0.25548 (7)	0.0143 (3)	
O2	0.62546 (18)	0.51362 (15)	0.38308 (8)	0.0167 (3)	
O3	0.33723 (18)	0.93937 (14)	0.19964 (8)	0.0163 (3)	
O4	0.17222 (18)	0.69382 (14)	0.14483 (8)	0.0144 (3)	
O5	0.6551 (2)	0.72932 (17)	0.55885 (9)	0.0201 (3)	
H5A	0.572 (4)	0.793 (3)	0.5817 (18)	0.053 (8)*	
H5B	0.748 (4)	0.733 (3)	0.5952 (17)	0.037 (8)*	
N1	0.0477 (2)	0.84612 (18)	0.33031 (9)	0.0150 (3)	
N2	0.2829 (2)	0.64620 (17)	0.43486 (9)	0.0136 (3)	
N3	0.7241 (2)	0.96452 (17)	−0.03740 (9)	0.0115 (3)	
H3A	0.7142	1.0023	−0.0898	0.014*	
N4	0.7962 (2)	1.02237 (18)	0.12227 (9)	0.0142 (3)	
N5	0.8275 (2)	1.23050 (17)	0.03349 (9)	0.0132 (3)	
H5C	0.8654	1.3024	0.0827	0.016*	
H5D	0.8193	1.2640	−0.0201	0.016*	
C1	0.3367 (2)	0.6769 (2)	0.35513 (11)	0.0111 (3)	
C2	0.2154 (3)	0.7745 (2)	0.30243 (11)	0.0114 (3)	
C3	−0.0026 (3)	0.8140 (2)	0.40958 (11)	0.0154 (4)	
H3	−0.1212	0.8619	0.4311	0.018*	
C4	0.1129 (3)	0.7127 (2)	0.46163 (11)	0.0150 (4)	
H4	0.0704	0.6903	0.5171	0.018*	
C5	0.5328 (2)	0.6005 (2)	0.32850 (11)	0.0115 (4)	
C6	0.2498 (2)	0.8028 (2)	0.20784 (11)	0.0123 (4)	
C7	0.7832 (2)	1.0727 (2)	0.03993 (11)	0.0115 (4)	
C8	0.7472 (3)	0.8626 (2)	0.12403 (12)	0.0157 (4)	
H8	0.7518	0.8251	0.1814	0.019*	
C9	0.6893 (3)	0.7459 (2)	0.04676 (12)	0.0158 (4)	
H9	0.6572	0.6322	0.0514	0.019*	
C10	0.6801 (2)	0.7997 (2)	−0.03562 (12)	0.0133 (4)	
C11	0.6237 (3)	0.6941 (2)	−0.12543 (12)	0.0174 (4)	
H11A	0.7309	0.7070	−0.1641	0.026*	
H11B	0.4936	0.7298	−0.1542	0.026*	
H11C	0.6096	0.5762	−0.1170	0.026*	
O21	0.9070 (2)	0.46146 (16)	0.19544 (9)	0.0162 (3)	
O22	0.9951 (2)	0.2292 (2)	0.31495 (9)	0.0216 (3)	
O23	0.6098 (2)	0.07554 (19)	0.35418 (10)	0.0228 (3)	
H21A	0.809 (4)	0.516 (3)	0.2147 (16)	0.039 (7)*	
H21B	0.987 (4)	0.530 (3)	0.1837 (15)	0.030 (6)*	
H22A	1.001 (4)	0.142 (4)	0.2846 (18)	0.054 (9)*	
H22B	0.973 (4)	0.300 (3)	0.2803 (18)	0.049 (8)*	
H23A	0.708 (4)	0.136 (3)	0.3438 (17)	0.048 (8)*	
H23B	0.544 (4)	0.039 (3)	0.3088 (19)	0.049 (9)*	

### Atomic displacement parameters (Å^2^)

**Table:**

	*U*^11^	*U*^22^	*U*^33^	*U*^12^	*U*^13^	*U*^23^
Co2	0.01444 (19)	0.0194 (2)	0.01064 (18)	0.00400 (14)	0.00278 (13)	0.00658 (13)
O1	0.0159 (6)	0.0177 (6)	0.0109 (6)	0.0020 (5)	0.0042 (5)	0.0054 (5)
O2	0.0164 (6)	0.0223 (7)	0.0138 (6)	0.0075 (5)	0.0041 (5)	0.0085 (5)
O3	0.0210 (7)	0.0146 (6)	0.0137 (6)	−0.0042 (5)	−0.0001 (5)	0.0054 (5)
O4	0.0175 (6)	0.0148 (6)	0.0108 (6)	−0.0018 (5)	0.0004 (5)	0.0026 (5)
O5	0.0173 (7)	0.0245 (8)	0.0181 (7)	0.0034 (6)	0.0006 (6)	0.0024 (6)
N1	0.0146 (7)	0.0163 (8)	0.0137 (7)	0.0023 (6)	0.0008 (6)	0.0018 (6)
N2	0.0142 (7)	0.0164 (8)	0.0105 (7)	0.0000 (6)	0.0017 (6)	0.0027 (6)
N3	0.0122 (7)	0.0132 (7)	0.0100 (7)	0.0011 (6)	0.0021 (6)	0.0040 (6)
N4	0.0136 (7)	0.0171 (8)	0.0127 (7)	0.0004 (6)	0.0016 (6)	0.0054 (6)
N5	0.0172 (8)	0.0141 (8)	0.0083 (7)	−0.0014 (6)	0.0001 (6)	0.0030 (6)
C1	0.0128 (8)	0.0110 (8)	0.0095 (8)	−0.0017 (6)	0.0006 (7)	0.0019 (6)
C2	0.0127 (8)	0.0095 (8)	0.0112 (8)	−0.0025 (6)	0.0005 (7)	0.0003 (6)
C3	0.0141 (9)	0.0179 (9)	0.0139 (9)	0.0023 (7)	0.0028 (7)	0.0002 (7)
C4	0.0153 (9)	0.0193 (9)	0.0109 (8)	0.0003 (7)	0.0042 (7)	0.0018 (7)
C5	0.0128 (8)	0.0101 (8)	0.0113 (9)	−0.0009 (7)	0.0003 (7)	0.0011 (7)
C6	0.0104 (8)	0.0145 (9)	0.0130 (9)	0.0041 (7)	0.0011 (7)	0.0050 (7)
C7	0.0067 (8)	0.0162 (9)	0.0120 (8)	0.0019 (7)	0.0019 (7)	0.0028 (7)
C8	0.0110 (8)	0.0228 (10)	0.0156 (9)	0.0029 (7)	0.0022 (7)	0.0102 (7)
C9	0.0130 (9)	0.0140 (9)	0.0217 (10)	0.0004 (7)	0.0023 (7)	0.0071 (7)
C10	0.0075 (8)	0.0145 (9)	0.0182 (9)	0.0014 (7)	0.0027 (7)	0.0027 (7)
C11	0.0189 (9)	0.0145 (9)	0.0185 (9)	0.0009 (7)	0.0032 (7)	0.0006 (7)
O21	0.0162 (7)	0.0146 (7)	0.0184 (7)	−0.0007 (6)	0.0054 (5)	0.0013 (5)
O22	0.0257 (8)	0.0183 (7)	0.0198 (7)	0.0030 (6)	0.0000 (6)	0.0018 (6)
O23	0.0199 (8)	0.0266 (8)	0.0198 (8)	−0.0015 (6)	0.0007 (6)	−0.0016 (6)

### Geometric parameters (Å, °)

**Table:**

Co2—O2^i^	2.0455 (12)	N5—H5C	0.8800
Co2—O2	2.0455 (12)	N5—H5D	0.8800
Co2—N2^i^	2.1101 (14)	C1—C2	1.390 (2)
Co2—N2	2.1101 (14)	C1—C5	1.517 (2)
Co2—O5^i^	2.1167 (14)	C2—C6	1.513 (2)
Co2—O5	2.1167 (14)	C3—C4	1.386 (2)
O1—C5	1.240 (2)	C3—H3	0.9500
O2—C5	1.263 (2)	C4—H4	0.9500
O3—C6	1.259 (2)	C8—C9	1.396 (2)
O4—C6	1.249 (2)	C8—H8	0.9500
O5—H5A	0.83 (3)	C9—C10	1.363 (2)
O5—H5B	0.77 (3)	C9—H9	0.9500
N1—C3	1.332 (2)	C10—C11	1.492 (2)
N1—C2	1.342 (2)	C11—H11A	0.9800
N2—C4	1.334 (2)	C11—H11B	0.9800
N2—C1	1.343 (2)	C11—H11C	0.9800
N3—C10	1.358 (2)	O21—H21A	0.85 (3)
N3—C7	1.361 (2)	O21—H21B	0.81 (2)
N3—H3A	0.8800	O22—H22A	0.79 (3)
N4—C8	1.325 (2)	O22—H22B	0.83 (3)
N4—C7	1.349 (2)	O23—H23A	0.85 (3)
N5—C7	1.318 (2)	O23—H23B	0.78 (3)
			
O2^i^—Co2—O2	180.0	C1—C2—C6	124.49 (15)
O2^i^—Co2—N2^i^	80.00 (5)	N1—C3—C4	121.94 (16)
O2—Co2—N2^i^	100.00 (5)	N1—C3—H3	119.0
O2^i^—Co2—N2	100.00 (5)	C4—C3—H3	119.0
O2—Co2—N2	80.00 (5)	N2—C4—C3	120.86 (16)
N2^i^—Co2—N2	180.000 (1)	N2—C4—H4	119.6
O2^i^—Co2—O5^i^	89.61 (5)	C3—C4—H4	119.6
O2—Co2—O5^i^	90.39 (5)	O1—C5—O2	126.40 (15)
N2^i^—Co2—O5^i^	86.62 (6)	O1—C5—C1	117.16 (14)
N2—Co2—O5^i^	93.38 (6)	O2—C5—C1	116.44 (14)
O2^i^—Co2—O5	90.39 (5)	O4—C6—O3	126.31 (15)
O2—Co2—O5	89.61 (5)	O4—C6—C2	116.05 (14)
N2^i^—Co2—O5	93.38 (6)	O3—C6—C2	117.32 (14)
N2—Co2—O5	86.62 (6)	N5—C7—N4	119.64 (15)
O5^i^—Co2—O5	180.00 (7)	N5—C7—N3	118.68 (15)
C5—O2—Co2	116.20 (10)	N4—C7—N3	121.68 (15)
Co2—O5—H5A	108.8 (19)	N4—C8—C9	124.00 (16)
Co2—O5—H5B	121.8 (18)	N4—C8—H8	118.0
H5A—O5—H5B	105 (3)	C9—C8—H8	118.0
C3—N1—C2	116.87 (15)	C10—C9—C8	118.16 (16)
C4—N2—C1	118.22 (14)	C10—C9—H9	120.9
C4—N2—Co2	130.55 (12)	C8—C9—H9	120.9
C1—N2—Co2	111.19 (11)	N3—C10—C9	117.89 (15)
C10—N3—C7	121.63 (14)	N3—C10—C11	116.07 (15)
C10—N3—H3A	119.2	C9—C10—C11	126.04 (16)
C7—N3—H3A	119.2	C10—C11—H11A	109.5
C8—N4—C7	116.59 (15)	C10—C11—H11B	109.5
C7—N5—H5C	120.0	H11A—C11—H11B	109.5
C7—N5—H5D	120.0	C10—C11—H11C	109.5
H5C—N5—H5D	120.0	H11A—C11—H11C	109.5
N2—C1—C2	120.13 (15)	H11B—C11—H11C	109.5
N2—C1—C5	116.11 (14)	H21A—O21—H21B	106 (2)
C2—C1—C5	123.75 (14)	H22A—O22—H22B	107 (3)
N1—C2—C1	121.91 (15)	H23A—O23—H23B	110 (3)
N1—C2—C6	113.51 (14)		

Symmetry codes: (i) −*x*+1, −*y*+1, −*z*+1.

### Hydrogen-bond geometry (Å, °)

**Table:**

*D*—H···*A*	*D*—H	H···*A*	*D*···*A*	*D*—H···*A*
N3—H3A···O3^ii^	0.88	1.77	2.6487 (17)	172
N5—H5C···O21^iii^	0.88	1.96	2.8350 (18)	172
N5—H5D···O4^ii^	0.88	1.96	2.8310 (18)	171
O21—H21B···O4^iv^	0.81 (2)	1.97 (3)	2.7777 (18)	175 (2)
O21—H21A···O1	0.85 (3)	1.86 (3)	2.7093 (18)	177 (2)
O5—H5B···O22^v^	0.77 (3)	2.02 (3)	2.784 (2)	172 (2)
O5—H5A···O23^i^	0.83 (3)	1.88 (3)	2.706 (2)	173 (3)
O23—H23B···O3^vi^	0.78 (3)	2.06 (3)	2.8302 (19)	172 (3)
O22—H22B···O21	0.83 (3)	1.97 (3)	2.794 (2)	177 (3)
O23—H23A···O22	0.85 (3)	2.13 (3)	2.959 (2)	165 (2)
O22—H22A···N1^vii^	0.79 (3)	2.59 (3)	3.152 (2)	130 (2)
O22—H22A···N4^vi^	0.79 (3)	2.68 (3)	3.242 (2)	130 (2)

Symmetry codes: (ii) −*x*+1, −*y*+2, −*z*; (iii) *x*, *y*+1, *z*; (iv) *x*+1, *y*, *z*; (v) −*x*+2, −*y*+1, −*z*+1; (i) −*x*+1, −*y*+1, −*z*+1; (vi) *x*, *y*−1, *z*; (vii) *x*+1, *y*−1, *z*.
